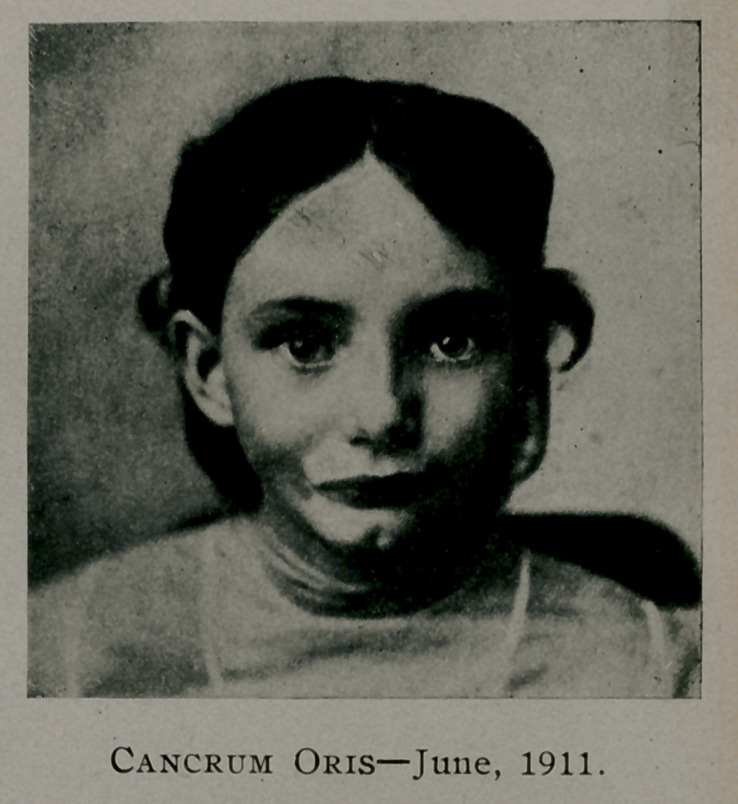# Radical Surgery in Cases of Cancrum-Oris or “Noma”, with Report of a Case of Bi-Lateral Infection Complicating Typhoid Fever with Recovery

**Published:** 1912-09

**Authors:** Baxter S. Moore, John S. Clifford

**Affiliations:** Atlanta, Ga.; Charlotte, N. C.


### Journal-Record of Medicine

Successor to Atlanta Medical and Surgical Journal, Established 1855
and Southern Medical Record, Established

OWNED BY THE ATLANTA MEDICAL JOURNAL CO.

Published Monthly

*Official Organ Fulton County Medical Society, State Examining Board, Presbyterian Hospital, Atlanta, Birmingham and Atlantic Railroad Surgeons' Association, Chattahoochee Valley Medical and Surgical Association, Etc.*

EDGAR G. BALLENGER, M. D., Editor.
BERNARD WOLFF, M. D., Supervising Editor.
A. W. STIRLING, M. D., C. M., D. P. H., J. S. HURT, B. Ph., M. D.
GEO. M. NILES, M. D., W. J. LOVE, M. D., (Ala.); Associate Editors.
E. W. ALLEN, Business Manager.

COLLABORATORS

Dr. W. F. WESTMORLAND, General Surgery.
F. W. McRAE, M. D., Abdominal Surgery.
H. F. HARRIS, M. D., Pathology and Bacteriology.
E. B. BLOCK, M. D., Diseases of the Nervous System.
MICHAEL HOKE, M. D., Orthopedic Surgery.
CYRUS W. STRICKLER, M. D., Legal Medicine and Medical Legislation.
E. C. DAVIS, A. B., M. D., Obstetrics.
E. G. JONES, A. B., M. D., Gynecology.
R. T. DORSEY, Jr., B. S. M. D., Medicine.
L. M. GAINES, A. B., M. D., Internal Medicine.
GEO. C. MIZELL, M. D., Diseases of the Stomach and Intestines.
L. B. CLARKE, M. D., Pediatrics.
EDGAR PAULIN, M. D., Opsonic Medicine.
THEODORE TOEPEL, M. D., Mechano Therapy.
R. R. DALY, M. D., Medical Society.
A. W. STIRLING, M. D., etc., Diseases of the Eye, Ear, Nose and Throat.
BERNARD WOLFF, M. D., Diseases of the Skin.
E. G. BALLENGER, M. D., Diseases of the Genito-Urinary Organs.

VOL. LIX. SEPTEMBER 1912 NO. 6

### RADICAL SURGERY IN CASES OF CANCRUM-ORIS OR "NOMA," WITH REPORT OF A CASE OF BI-LATERAL INFECTION COMPLICATING TYPHOID FEVER WITH RECOVERY.

BY BAXTER S. MOORE, M. D., OF ATLANTA, AND DR. JOHN S. CLIFFORD OF CHARLOTTE, N. C.

Only until recent years were the surgical complications arising during a typhoid attack given the consideration that is their due, and it remained for Keen, in his excellent monograph upon this subject, to call the attention of the profession to the manifold conditions that bring the surgeon to the bedside of the typhoid patient.

Gangrene, while comparatively rare, is an interesting complication and in the 133 cases collected from the literature by Keen there were nine cases of Noma, five of these terminated fatally, in one case the final result was not recorded. The single case of both cheeks being involved, originally reported by Littlejohn, ended in death.

Most of the text-books dwell but lightly upon this subject, simply stating that it is a progressive gangrenous process beginning on the mucus membrane of the cheek or gums and spreading rapidly to the cutaneous surface. It is usually observed in children between the ages of three and eight, complicating some acute infectious fever, more often measles than any other, and is less common where proper attention is paid to hygiene. The pathology is not well worked out, as the disease is so rapid that limited time for study is afforded. In our case a positive Widal was reported, and no doubt the Bacillus of Eberth was the principal invading organism. We regret that no culture was taken. The treatment, however, is well defined and the only efficient measure to pursue is the total destruction of the tissues involved by the actual cautery, even extending well into the surrounding healthy tissues seemingly without any regard for the cosmatic results. As in the case reported herein one application is rarely sufficient, but at the slightest evidence of involvement of any new areas recourse must be had to the thermo cautery.

We report the following case because of the interesting fact that it was a bi-lateral infection and also on account of the invasion of the superior maxilla, on the right side of the antrum was entered, while on the left side the alveolar processes of two molar teeth were destroyed. It is also interesting to note that on the right side the orifice of Stensons duct was obliterated, yet there was no involvement of glandular tisue.

Report of Case.—M. C., aged 8, family history negative, youngest child of widowed mother who lived in a mill settlement, had been ill at home with typhoid fever for three weeks. Was sent into the Mercy hospital by Dr. McManaway, August 13, 1910, with a temperature of 103.4, pulse 163, respiration 30, and for

Cancrum Oris—August, 1910.

Cancrum Oris—November, 1910.

Cancrum Oris—June, 1911.

several days previous to admission had had involuntary evacuations from both the bladder and rectum. The child was semi-conscious and showed a great lack of proper care, there being absolutely no attention paid to her personal cleanliness. The patient came under our observation August 19, and upon examination a well defined gangrenous area (figure 1) was seen on the right cheek, which was said to have appeared as a small reddish blue spot, five days previous. The patient was at once placed under ether anaesthesia and the infected area treated with the actual cautery, two molar teeth, which were loose were extracted and their alveolar processes came out with them. It was then observed that the antrum was involved. This was curetted, packed with gauze saturated in alcohol and the patient returned to bed. The dressings were renewed every three hours and the proper nourishment and stimulation administered. The patient reacted nicely, the temperature came down and there was no incontinence of either urine or feces. On August 22nd it was necessary to apply the cautery to the edges of the wound, the antrum gave no trouble and the patient commenced to complain of the pain following the change of the dressings, which were soaked in alcohol. On August 25th at 6 P. M., a small reddish area, about the size of a five-cent piece was observed on the left cheek and immediately the cheek was incised down to and through the orbicularies muscle (figure 2) the actual cautery was applied to the edges of this wound and two molar teeth, which were loose, extracted, and the alveolar processes as on the right side were removed. The patient emerged from the anaesthetic very delirious and the condition persisted for five days. There was a marked rise in temperature 104.4, pulse 180 with great prostration. On August 29th, and again on September 4th, it was necessary to apply the cautery to the sloughing edges of the wound on the right cheek. The temperature remained high, with daily remissions from one to two and one-half degrees until September 11th, when a well-marked curve of defervescence began until normal was maintained from October 6th. The wound on the left cheek healed rapidly, the fibres of the orbicularis oris were joined practically by primary union, the destruction on the right side was sufficient to restrict the movement of the lower jaw, due to a marked retraction of the remaining mucus membrane. The patient was sent home November 20th, 1910, and her mother promised to have her return to the hospital in six months in order to correct as much as possible the deformity on the right side. The child gained in weight rapidly and was sent to school in the spring of 1911. When last seen, October, 1911 (figure 3), the child could open her mouth about one-half an inch and her mother refused to have her submit to the plastic operation necessary to further increase the movement of the inferior maxilla.

Respectfully submitted,
JOHN S. CLIFFORD, M. D.,
BAXTER S. MOORE, M. D.

## Figures and Tables

**Figure f1:**
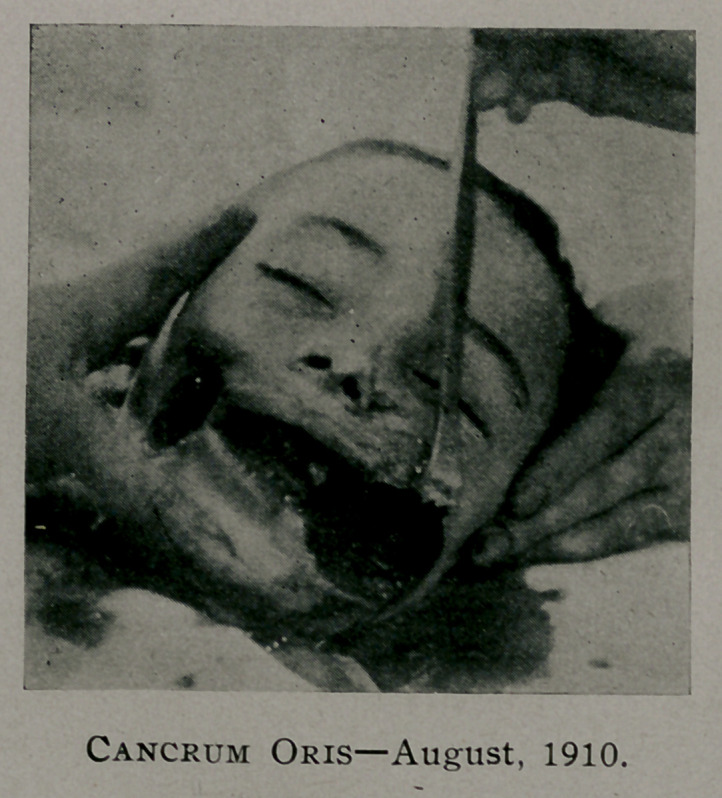


**Figure f2:**
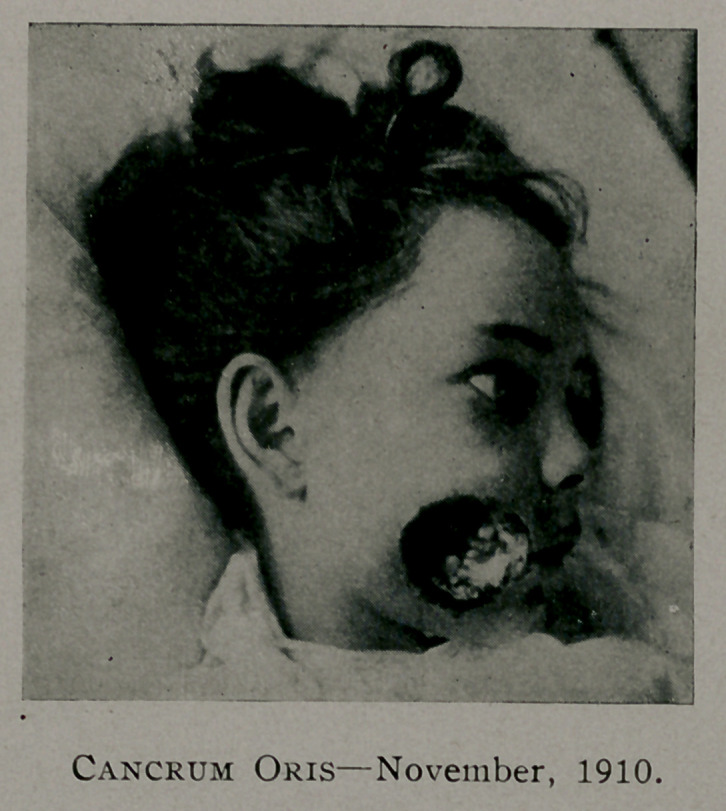


**Figure f3:**